# Comparison of cecal microbiota composition in hybrid pigs from two separate three-way crosses

**DOI:** 10.5713/ab.20.0681

**Published:** 2020-12-01

**Authors:** Yuting Yang, Liyan Shen, Huan Gao, Jinming Ran, Xian Li, Hengxin Jiang, Xueyan Li, Zhenhui Cao, Ying Huang, Sumei Zhao, Chunlian Song, Hongbin Pan

**Affiliations:** 1 Yunnan Provincial Key Laboratory of Animal Nutrition and Feed Science, Faculty of Animal Science and Technology, Yunnan Agricultural University, Kunming 650201, China; 2 Dazhou Vocational and Technical College, Dazhou 635000, China; 3 Collge of Veterinary Medicine, Yunnan Agricultural University, Kunming 650201, China

**Keywords:** Saba Pig, Duroc Pig, Berkshire Pig, Three-way Cross Model, Cecal Microbiota, 16S rRNA Sequencing

## Abstract

**Objective:**

The intestinal microbiota plays an important role in host physiology, metabolism, immunity, and behavior. And host genetics could influence the gut microbiota of hybrid animals. The three-way cross model is commonly utilized in commercial pig production; however, the use of this model to analyse the gut microbial composition is rarely reported.

**Methods:**

Two three-way hybrid pigs were selected, with Saba pigs as the starting maternal pig: Duroc× (Berkshire×Saba) (DBS) pig, Berkshire×(Duroc×Saba) (BDS) pig. One hundred pigs of each model were reared from 35 days (d) to 210 d. The body weight or feed consumption of all pigs were recorded and their feed/gain (F/G) ratio was calculated. On day 210, 10 pigs from each three-way cross were selected for slaughter, and cecal chyme samples were collected for 16S rRNA gene sequencing.

**Results:**

The final body weight (FBW) and average daily gain (ADG) of DBS pigs were significantly higher than those of BDS pigs (p<0.05), while the F/G ratios of DBS pigs were significantly lower than those of BDS pigs (p<0.05). The dominant phyla in DBS and BDS pigs were Bacteroidetes (55.23% vs 59%, respectively) and Firmicutes (36.65% vs 34.86%, respectively) (p>0.05). At the genus level, the abundance of *Prevotella*, *Roseburia*, and *Anaerovibrio* in DBS pigs was significantly lower than in BDS pigs (p<0.01). The abundance of *Eubacterium*, *Clostridium XI*, *Bacteroides*, *Methanomassiliicoccus*, and *Parabacteroides* in DBS pigs was significantly higher than in BDS pigs (p<0.05). The FBWs and ADGs were positively correlated with *Bacteroides*, *ClostridiumXI*, and *Parabacteroides* but negatively correlated with the *Prevotella*, *Prevotella*/Bacteroides (P/B) ratio, *Roseburia*, and *Anaerovibrio*.

**Conclusion:**

These results indicated that host genetics affect the cecal microbiota composition and the porcine gut microbiota is associated with growth performance, thereby suggesting that gut microbiota composition may be a useful biomarker in porcine genetics and breeding.

## INTRODUCTION

The intestinal microbiota is a complex ecosystem that plays a major role in the physiology and health of the host [1]. Studies have found that host genetics impact the composition of gut microbiota and their metabolites [2]. Pig is one of the most important economic animals and an ideal model for studying human physiological function and disease [3]. Pigs of different breeds have phenotypically different genetic makeups that result in diverse physiological traits as well as gastrointestinal microbiomes [4]. The Saba pig is a traditional native pig breed found in Chuxiong Autonomous Prefecture, Yunnan Province, and has a high reproductive rate, good meat quality, and the capacity for high utilization of coarse feed [5]. However, large-scale domestic commercial farms rarely feed the local breed because of their unfavorable characteristics, which include slow growth rate and low lean meat rate. Cross breeding has been widely used in pig breeding because of pig heterosis and breed complementarity [6].

Commercial pig producers generally use a terminal crossbreeding system with three breeds, and, at present, a three-way cross model is largely used in commercial pig production [7]. Among the pigs involved in this model, Duroc pigs and Berkshire pigs are world-renowned lean pig breeds and are the main terminal sire breeds in commercial pig production [8].

An investigation into the intestinal microbial composition of hybrid pigs would reveal and promote the utilization of the genetic characteristics of different pig breeds. Thus, in the present study, we performed 16S rRNA gene sequencing analysis of the gut microbiomes of Duroc×(Berkshire×Saba) (DBS) pig and Berkshire×(Duroc×Saba) (BDS) pigs. Our aim was to characterize the differences in gut microbiome composition between these two hybrids and evaluate the underlying association of the gut microbiota with growth performance.

## MATERIALS AND METHODS

### Animal ethics statement

All animal works performed were approved by the Institutional Animal Care and Use Committee of the Yunnan Agricultural University (No. YNAU20181006). The care and use of animals fully complied with local animal welfare laws, guidelines and policies.

### Experimental animal and sample preparation

The experiment was carried out on a commercial farm in the city of Kunming, China. We selected 100 35-day (d)-old DBS and 100 BDS three-way hybrid weaned piglets (half males and half females), and all piglets were raised in the same temperature-controlled house, with 10 piglets per pen and 10 pens per treatment (length 4 m, width 3 m). There is a central passage with pens on either side, the pen floor was made of concrete and the house is naturally ventilated. Pigs were fed a three-stage NRC diet (Table 1), and were allowed free access to water and were *ad libitum* fed till 210 d.

At the end of the 210-day trial, 20 pigs (ten from each group) were sacrificed by exsanguination. Then the cecal chyme samples were collected into sterile tubes and quick-frozen in liquid nitrogen before stored at −80°C for 16S rRNA analysis. Body weight gain, feed consumption, and the feed/gain (F/G) ratio for these pigs were recorded from 35 d to 210 d. Polymerase chain reaction amplification, 16S rRNA amplicon sequencing, and processing of sequence data were performed as previously described [9].

### Statistical analysis

Experimental data, including those for growth performance and microbial abundance, were analyzed using the SPSS 22.0 software (IBM SPSS Statistics for Windows; IBM Corp, NY, USA). The Shapiro-Wilk test was used to evaluate normality. The general linear model of the Duncan multiple comparison test was used to analyze the parameter data, and Kruskal-Wallis analysis of variance was used for the phylum and genus level microbiota. Growth performance and microbial data are expressed as the mean±standard error. Phylogenetic studies of the PICRUSt communities were based on operational taxonomic units (OTUs) [10]. To assess the correlation between dominant genera and growth performance, we performed Spearman’s test in GraphPad Prism 7.0 [11]. A p-value <0.05 was considered to be statistically significant.

## RESULTS

### Comparison of growth performances of DBS and BDS hybrid pigs

The final body weight (FBW) and average daily gain (ADG) of DBS pigs were significantly higher compared to those of BDS pigs, while the F/G ratio of DBS pigs was significantly lower than that of BDS pigs (p<0.05; Table 2).

### Changes in the diversity of the microbial community in the cecum

**Figure 1:**
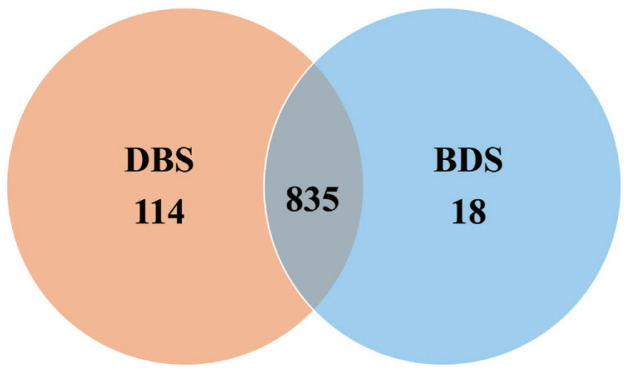
Venn diagram of microbial OTUs clustered at 97% sequence identity in DBS and BDS pigs. The overlapping area represent shared OTUs numbers between different pigs, in which 835 core OTUs are identified in the two groups, and 114 and 18 unique OTUs are identified in the DBS and BDS groups. OTUs, operational taxonomic units; DBS, Duroc×(Berkshire×Saba); BDS, Berkshire×(Duroc×Saba).

A total of 20 cecal samples generated 1,209,608 clean reads, with an average of 60,480 clean reads per sample and an average clean read length of 415 bp. OTUs were obtained at a sequence similarity level of 97%. A Venn diagram was used to reveal the shared and unique microbial percentages in the DBS and/or BDS groups. Additionally, 835 core OTUs were identified in the two groups, and 114 and 18 unique OTUs were identified in the DBS and BDS groups, respectively (Figure 1).

**Figure 2:**
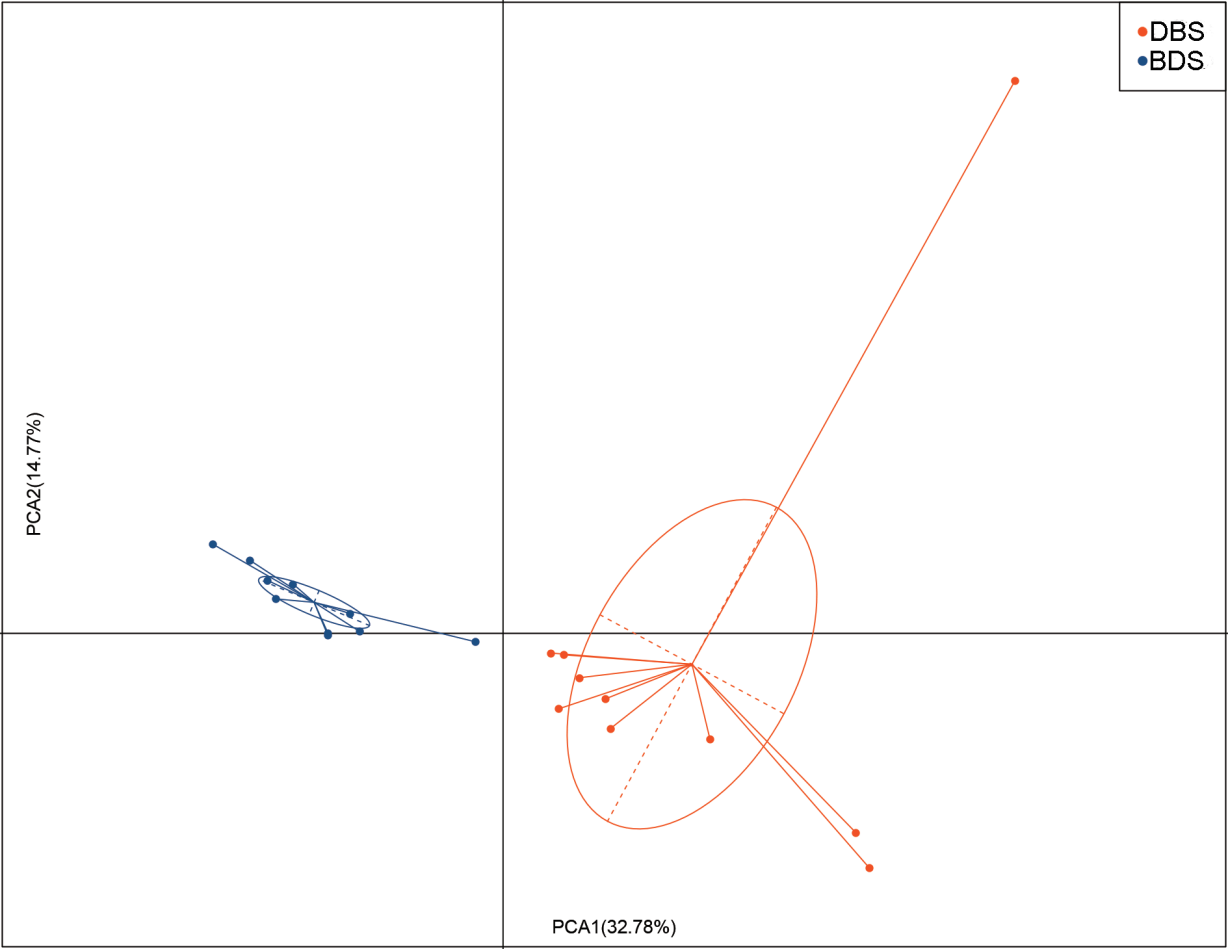
Principal component analysis (PCA) of cecal microbial beta-diversity in DBS and BDS pigs (red denotes the DBS group and blue denotes the BDS group). The percentage of the variation explained by the plotted principal coordinates is indicated on the axes. DBS, Duroc×(Berkshire×Saba); BDS, Berkshire×(Duroc×Saba).

Diversity indices were calculated based on the OTUs of each library. The Chao1 index, phylogenetic diversity whole tree index, good’s coverage, Shannon index, observed species indices, and Simpson index were used to evaluate the abundance and diversity of the microbial species in the samples. We found that there were differences in the alpha diversity indices between DBS and BDS pigs, but the differences were not statistically significant (Table 3). Principal component analyses (PCA) were used to estimate the beta diversity between the two groups. The PCA plot of the unweighted unifrac distances showed that the DBS and BDS groups had a distinct difference (p<0.05; Figure 2).

### Microbial composition in the ceca of DBS pigs and BDS pigs

At the phylum level, 16 and 14 taxa were identified in DBS and BDS pigs, respectively. The dominant phyla of the two groups were Bacteroidetes, Firmicutes, Spirochetes, and Proteobacteria, and the abundances of these 4 dominant phyla in DBS pigs were 55.23%, 36.65%, 2.7%, and 2.86%, respectively. Similarly, the abundances of these 4 dominant phyla in the BDS pigs were 59%, 34.86%, 2.92%, and 2.27%, respectively (Table 4).

Twenty-one genera were identified in the DBS and BDS groups. The abundances of *Prevotella*, *Roseburia*, and *Anaerovibrio* in DBS pigs were significantly lower than those in BDS pigs (p<0.01). The abundances of *Bacteroides*, *Methanomassiliicoccus*, and *Parabacteroides* in DBS pigs were significantly higher than those in BDS pigs (p<0.05). The abundances of *Eubacterium* and *Clostridium XI* in DBS pigs were significantly higher than those in BDS pigs (p<0.01; Table 5).

**Figure 3:**
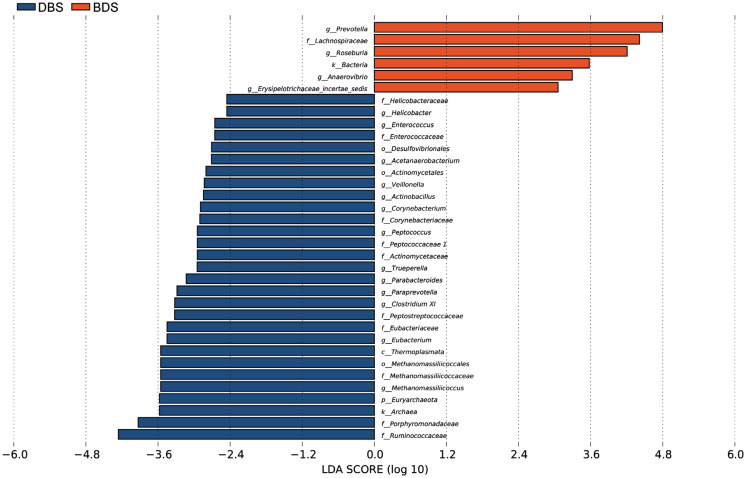
Linear discriminant analysis effect size (LEfSe) analysis based on OTUs characterizes microbiota between DBS and BDS pigs. Histogram of the linear discriminant analysis (LDA) scores computed for features differentially abundant (significant threshold>2 fold and p<0.05) among pigs was shown. OTUs, operational taxonomic units; DBS, Duroc×(Berkshire×Saba); BDS, Berkshire×(Duroc×Saba).

To determine the specific bacterial taxonomic groups associated with different hybrid pigs, we performed a linear discriminant analysis effect size (LEfSe) to compare the cecal microbiotas of DBS and BDS pigs. The data showed that at the kingdom level, Archaea was significantly enriched in the DBS group, and Bacteria were significantly enriched in the BDS group (Figure 3). At the phylum level, Desulfovibrionales was significantly enriched in the DBS group. At the class level, Thermoplasmata was significantly enriched in the DBS group. At the order level, Methanomassilicoccales, Desulfovibrionales, and Actinomycetales were significantly enriched in the DBS group. At the family level, there was significant enrichment of Helicobacteraceae, Enterococcaceae, Corynebacteriaceae, Methanomassiliicoccaceae, Porphyromonadaceae, Ruminococcaceae, Peptococcaceae I, Actinomycetaceae, Peptostreptococcaceae, and Eubacteriaceae in the DBS group and that of Lachnospiraceae in the BDS group. At the genus level, *Helicobacter*, *Enterococcus*, *Acetanaerobacterium*, *Veillonella*, *Actinobacillus*, *Corynebacterium*, *Eubacterium*, *Methanomassiliicoccus*, *Peptococcus*, *Trueperella*, *Parabacteroides*, *Paraprevotella*, and *Clostridium XI* were significantly enriched in the DBS group, and *Prevotella*, *Roseburia*, *Anaerovibrio*, and *Erysipelotrichaceae incertae sedis* were significantly enriched in the BDS group.

### Correlation between microbiota and growth performance

**Figure 4:**
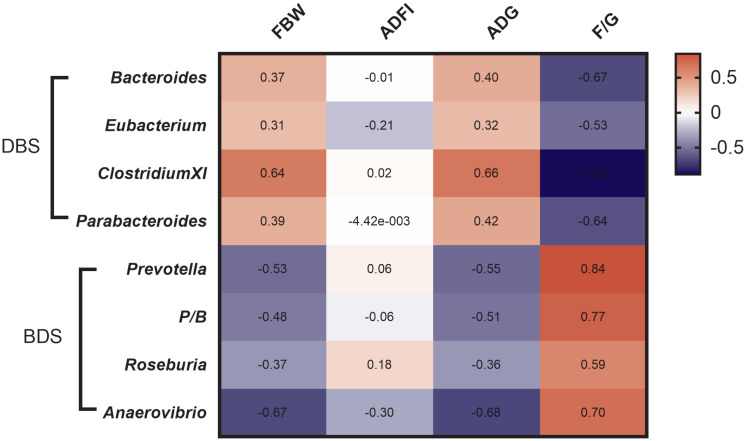
Heatmap of correlation between differential genera of cecal microbes and growth performance of pigs. Spearman’s test was used to calculate correlation coefficient. The negative correlation was expressed by blue color, the positive correlation was expressed by red color. FBW, final body weight; ADFI, average daily feed intake; ADG, average daily gain; F/G, the ratio of feed gain.

To investigate the correlation between cecal microbiota and the growth performance of these two hybrid pig models, a heat map was constructed (Figure 4). Our data showed that the FBW and ADG were positively correlated with *Bacteroides*, *ClostridiumXI*, and *Parabacteroides* but negatively correlated with *Prevotella*, *Prevotella/Bacteroides* (P/B) ratio, *Roseburia*, and *Anaerovibrio*. The F/G ratio was positively correlated with *Bacteroides*, *Eubacterium*, *ClostridiumXI*, and *Parabacteroides* but negatively correlated with *Prevotella*, P/B ratio, *Roseburia*, and *Anaerovibrio*.

## DISCUSSION

In the modern pig industry, crossbreeding is an effective method to improve the efficiency of and profit from production [12]. The Berkshire breed has excellent meat quality features, such as thin muscle fiber and excellent water holding capacity [13], the Duroc breed has both excellent growth rate and intramuscular fat and is used as a terminal sire when fattening pigs are produced [14]. Previous studies have reported that the growth rate of pigs produced by crossing DDL (Duroc×(Duroc×Landrace) and BDL (Berkshire×(Duroc× Landrace) pigs was higher than those of pure-breed Berkshire and Duroc pigs [15]. Studies have also shown that the ADG of Hampshire and Landrace hybrid pigs increased significantly and that the growth performance of hybrid pigs was higher than that of purebred pigs [16]. Further, studies have found that hybrid pigs show superior genetics at the production level [17]. The Saba pig is one of the most important local pig breeds in Yunnan Province and the main maternal breed used in hybrid systems in central Yunnan. Our results show that the FBW and ADG of DBS pigs were significantly higher than those of BDS pigs, while the F/G ratio of DBS pigs was significantly lower than that of BDS pigs. These data show that DBS pigs have a better growth performance than BDS pigs.

Intestinal microbiota are generally thought to play a major role in bodily functions and have a considerable impact on the growth and health of the host [18,19]. Furthermore, studies have shown that host genetics have a significant impact on the composition of the intestinal microbiome [20]. Moreover, the type of pig breed is a major factor affecting the intestinal microbial composition. An investigation of the jejunal and colonic microbial communities in pure-breed Meishan and Yorkshire piglets showed that the type of breed has a significant impact on the bacterial community structure on days 14 and 49 [21]. In addition, the importance of host genetics in shaping the gastrointestinal microbiota in hybrids has also been demonstrated in mouse models. For instance, researchers found that hybrid mice displayed widespread transgressive phenotypes in their bacterial communities, which exhibited a structure associated with aberrant immune gene expression and increased intestinal pathology [22]. Therefore, host genetics also have a major impact on the microbiome and metabolites in hybrid animals [2]. Our data showed that the gut microbiota differed between two related hybrid pigs, with differences existing in some genera; no difference existed in the microbial diversity between DBS and BDS pigs, suggesting that hybridization has a major effect on the cecal microbiota composition at the genus level.

Many studies on intestinal microbiota have shown that Firmicutes and Bacteroides are the two most dominant phyla in the animal intestine [23], which is consistent with our work showing that the dominant intestinal microbes in the two hybrid pigs were Firmicutes and Bacteroides at the phylum level. *Bacteroides* and *Prevotella* are the two main genera in the Bacteroidetes phylum [24]. *Prevotella* ferments complex polysaccharides from the diet to produce succinate [25]. *Bacteroides* produce propionate and harvest energy more efficiently from food than commensal gut microbes [19,26]. Additionally, high fat and protein intake are associated with increased levels of *Bacteroides*, while high fiber intake is associated with increased levels of *Prevotella* [24]. A previous study showed that overweight pregnant women had more *Bacteroides-Prevotella* group bacteria than normal-weight pregnant women [27]. Individuals with a high P/B ratio lost more body weight and body fat than individuals with a low P/B ratio, confirming that individuals with a high P/B ratio are more susceptible to weight loss [28]. Our results showed that FBW and ADG are positively correlated with *Bacteroides* and negatively correlated with *Prevotella* and P/B ratio, which agrees with previous studies. These observations suggest that the P/B ratio may be considered as an important biomarker in weight management as well as pig breeding.

Taken together, the experimental findings presented in this study show that DBS pigs have better growth performance than BDS pigs. In addition, differences existed in some genera, with no difference in diversity between DBS and BDS pigs. Furthermore, our study provides evidence that that host genetics affect the cecal microbiota composition and the porcine gut microbiota is associated with growth performance, thereby suggesting that gut microbiota composition may be a useful biomarker in porcine genetics and breeding.

## Figures and Tables

**Table 1:** Diet compositions (as fed basis)

Items	Phase 1 (35 to d)	Phase 2 (101 to d)	Phase 3 (162 to d)
Ingredient (%)			
Yellow corn	60.26	60.63	62.29
Wheat bran	3	11.14	13.95
Soybean oil	1	2.1	2.5
Extruded soybean meal	12.4	15.8	11.76
Fermented soybean meal	15	7	6
Fish meal	4	0	0
L-lyssine-HCl	0.8	0.33	0.65
DL-methionine	0.04	0.05	0.1
Limestone	0.89	1.12	0.98
CaHPO_4_	1.31	0.48	0.42
NaCl	0.3	0.35	0.35
Premix	1^1)^	1^2)^	1^3)^
Total	100	100	100
Calculated composition (%)			
Digestible energy (MJ/kg)	14.56	14.27	14.25
Crude protein	22.75	18.34	16.76
Ca	0.91	0.62	0.54
Total P	0.76	0.53	0.51
Lys	1.81	1.14	1.25
Met+Cys	0.79	0.73	0.75

1) Supplied per kg diet: 140 mg Fe, 80 mg Zn, 110 mg Cu, 60 mg Mn, 0.26 mg I, 0.57 mg Se, 8,500 IU vitamin A, 3,750 IU vitamin D_3_, 21 IU vitamin E, 4.5 mg vitamin K_3_, 4 mg vitamin B_1_, 10 mg vitamin B_2_, 4.28 mg vitamin B_6_, 28 μg vitamin B_12_, 37 mg niacin, 15.4 mg pantothenic acid, 0.15 mg biotin.

2) Supplied per kg diet: 120 mg Fe, 50 mg Zn, 14 mg Cu, 50 mg Mn, 0.25 mg I, 0.3 mg Se, 7,500 IU vitamin A, 2,200 IU vitamin D_3_, 30 IU vitamin E, 2.6 mg vitamin K_3_, 2.6 mg vitamin B_1_, 7.2 mg vitamin B_2_, 4.28 mg vitamin B_6_, 27 μg vitamin B_12_, 30.3 mg niacin, 13.8 mg pantothenic acid, 0.11 mg biotin.

3) Supplied per kg diet: 80 mg Fe, 22 mg Zn, 14 mg Cu, 13 mg Mn, 0.25 mg I, 0.3 mg Se, 6,000 IU vitamin A, 1,500 IU vitamin D_3_, 22 IU vitamin E, 1.8 mg vitamin K_3_, 1.54 mg vitamin B_1_, 5.5 mg vitamin B_2_, 3.73 mg vitamin B_6_, 19 μg vitamin B_12_, 26.31 mg niacin, 9.4 mg pantothenic acid, 0.09 mg biotin.

**Table 2:** Growth performance of DBS^1)^ and BDS^1)^ pigs

Group ID	DBS	BDS	SEM	p-value
FBW (kg)	114^a^	104.94^b^	3.51	0.022
ADFI (kg)	1.77	1.78	0.036	0.906
ADG (kg)	0.6^a^	0.55^b^	0.02	0.027
F/G	2.95^b^	3.22^a^	0.055	<0.001

Values reported as means (n = 10).

SEM, standard error of means for 10 pigs each; FBW, final body weight; ADFI, average daily feed intake; ADG, average daily gain; F/G, the ratio of feed gain.

1) DBS, Duroc×(Berkshire×Saba); BDS, Berkshire×(Duroc×Saba).

a,b Means in the same row with different superscripts differ statistically (p<0.05).

**Table 3:** The alpha diversity of cecal microbial community in DBS^1)^ and BDS^1)^ pigs

Group ID	DBS	BDS	SEM	p-value
Chao1	663.73	611.81	36.29	0.175
Observed species	586.13	537.13	34.41	0.176
Phylogenetic diversity whole tree	39.48	36.85	1.84	0.174
Shannon	6.83	6.62	0.19	0.293
Simpson	0.97	0.97	0.0047	0.234
Goods coverage	0.9974	0.9975	0.00012	0.588

Values reported as means (n = 10).

SEM, standard error of means for 8 pigs each.

1) DBS, Duroc×(Berkshire×Saba); BDS, Berkshire×(Duroc×Saba).

**Table 4:** The relative abundances of dominant phyla of cecal microbes in DBS^1)^ and BDS^1)^ pigs

Tax name	DBS	BDS	SEM	p-value
Bacteroidetes	55.23	59	3.52	0.302
Firmicutes	36.65	34.86	2.12	0.415
Spirochaetes	2.7	2.92	1.18	0.855
Proteobacteria	2.86	2.27	0.51	0.265
Euryarchaeota	1.15	0.17	0.51	0.074

Values reported as means (n = 8).

SEM, standard error of means for 8 pigs each.

1) DBS, Duroc×(Berkshire×Saba); BDS, Berkshire×(Duroc×Saba).

**Table 5:** The relative abundances of differential genera of cecal bacteria in DBS^1)^ and BDS^2)^ pigs

Tax name	DBS	BDS	SEM	p-value
*Prevotella*	19.17^b^	39.87^a^	2.85	<0.001
*Roseburia*	4.54^b^	9.94^a^	1.24	0.001
*Bacteroides*	7.82^a^	2.80^b^	1.27	0.002
*Eubacterium*	2.21^a^	0.56^b^	0.64	0.022
*Clostridium XI*	1.84^a^	0.86^b^	0.32	0.008
*Parabacteroides*	1.26^a^	0.65^b^	0.26	0.032
*Anaerovibrio*	0.38^b^	0.94^a^	0.15	0.002

Values reported as means (n = 8).

SEM, standard error of means for 8 pigs each.

1) DBS, Duroc×(Berkshire×Saba); BDS, Berkshire×(Duroc×Saba).

a,b Means in the same row with different superscripts differ statistically (p<0.05).

## References

[1] Clemente JC, Ursell LK, Parfrey LW, Knight R (2012). The impact of the gut microbiota on human health: an integrative view. Cell.

[2] Li Z, Wright AG, Si H (2016). Changes in the rumen microbiome and metabolites reveal the effect of host genetics on hybrid crosses. Environ Microbiol Rep.

[3] Yang H, Xiao Y, Wang J (2018). Core gut microbiota in Jinhua pigs and its correlation with strain, farm and weaning age. J Microbiol.

[4] Pajarillo EA, Chae JP, Balolong MP, Kim HB, Seo KS, Kang DK (2014). Pyrosequencing-based analysis of fecal microbial communities in three purebred pig lines. J Microbiol.

[5] Diao S, Huang S, Chen Z (2019). Genome-wide signatures of selection detection in three south China indigenous pigs. Genes (Basel).

[6] Christensen OF, Legarra A, Lund MS, Su G (2015). Genetic evaluation for three-way crossbreeding. Genet Sel Evol.

[7] Kuhlers DL, Jungst SB, Little JA (1994). An experimental comparison of equivalent terminal and rotational crossbreeding systems in swine: pig performance. J Anim Sci.

[8] Suzuki K, Shibata T, Kadowaki H, Abe H, Toyoshima T (2003). Meat quality comparison of Berkshire, Duroc and crossbred pigs sired by Berkshire and Duroc. Meat Sci.

[9] Li X, Cao Z, Yang Y (2019). Correlation between jejunal microbial diversity and muscle fatty acids deposition in broilers reared at different ambient temperatures. Sci Rep.

[10] Javurek AB, Spollen WG, Ali AM (2016). Discovery of a novel seminal fluid microbiome and influence of estrogen receptor alpha genetic status. Sci Rep.

[11] Mitteer DR, Greer BD, Fisher WW, Cohrs VL (2018). Teaching behavior technicians to create publication-quality, single-case design graphs in graphpad prism 7. J Appl Behav Anal.

[12] Tang G, Yang R, Xue J (2013). Optimising a crossbreeding production system using three specialised imported swine breeds in south-western China. Anim Prod Sci.

[13] Ryu YC, Choi YM, Lee SH (2008). Comparing the histochemical characteristics and meat quality traits of different pig breeds. Meat Sci.

[14] Jang D, Yoon J, Taye M (2018). Multivariate genome-wide association studies on tenderness of Berkshire and Duroc pig breeds. Genes Genomics.

[15] Suzuki K, Shibata T, Kadowaki H, Abe H, Toyoshima T (2003). Meat quality comparison of Berkshire, Duroc and crossbred pigs sired by Berkshire and Duroc. Meat Sci.

[16] Baas TJ, Christian LL, Rothschild MF (1992). Heterosis and recombination effects in Hampshire and Landrace swine: I. Maternal traits. J Anim Sci.

[17] Godinho RM, Bergsma R, Silva FF (2018). Genetic correlations between feed efficiency traits, and growth performance and carcass traits in purebred and crossbred pigs. J Anim Sci.

[18] Reid G (2004). When microbe meets human. Clin Infect Dis.

[19] Turnbaugh PJ, Ley RE, Mahowald MA, Magrini V, Mardis ER, Gordon JI (2006). An obesity-associated gut microbiome with increased capacity for energy harvest. Nature.

[20] Davenport ER (2016). Elucidating the role of the host genome in shaping microbiome composition. Gut Microbes.

[21] Mu C, Bian G, Su Y, Zhu W (2019). Differential effects of breed and nursing on early-life colonic microbiota and immune status as revealed in a cross-fostering piglet model. Appl Environ Microbiol.

[22] Wang J, Kalyan S, Steck N (2015). Analysis of intestinal microbiota in hybrid house mice reveals evolutionary divergence in a vertebrate hologenome. Nat Commun.

[23] Crespo-Piazuelo D, Migura-Garcia L, Estellé J (2019). Association between the pig genome and its gut microbiota composition. Sci Rep.

[24] Kovatcheva-Datchary P, Nilsson A, Akrami R (2015). Dietary Fiber-induced improvement in glucose metabolism is associated with increased abundance of *Prevotella*. Cell Metab.

[25] Rampelli S, Schnorr SL, Consolandi C (2015). Metagenome sequencing of the Hadza hunter-gatherer gut microbiota. Curr Biol.

[26] Macy JM, Ljungdahl LG, Gottschalk G (1978). Pathway of succinate and propionate formation in *Bacteroides fragilis*. J Bacteriol.

[27] Collado MC, Isolauri E, Laitinen K, Salminen S (2008). Distinct composition of gut microbiota during pregnancy in overweight and normal-weight women. Am J Clin Nutr.

[28] Hjorth MF, Blædel T, Bendtsen LQ (2019). *Prevotella*-to-*Bacteroides* ratio predicts body weight and fat loss success on 24-week diets varying in macronutrient composition and dietary fiber: results from a post-hoc analysis. Int J Obes.

